# Estimation of the Disc Damage Likelihood Scale in primary open-angle glaucoma: the Glaucoma Stereo Analysis Study

**DOI:** 10.1007/s00417-015-3239-0

**Published:** 2015-12-15

**Authors:** Yasushi Kitaoka, Masaki Tanito, Yu Yokoyama, Koji Nitta, Maki Katai, Kazuko Omodaka, Toru Nakazawa

**Affiliations:** Department of Ophthalmology, St. Marianna University School of Medicine, 2-16-1 Sugao Miyamae-ku, Kawasaki, Kanagawa 216-8511 Japan; Division of Ophthalmology, Matsue Red Cross Hospital, 200 Horomachi, Matsue, Shimane 690-8506 Japan; Department of Ophthalmology, Shimane University Faculty of Medicine, Enya 89-1, Izumo, Shimane 693-8501 Japan; Department of Ophthalmology, Tohoku University Graduate School of Medicine, 1-1 Seiryo-machi, Aoba-ku, Sendai, 980-8574 Japan; Department of Ophthalmology, Fukui-ken Saiseikai Hospital, 7-1 Funabashi, Wadanaka-machi, Fukui 918-8503 Japan; Department of Ophthalmology, Sapporo Teishin Hospital, 14-Jo 1-5-1 Kawazoe, Minami-ku, Sapporo, 005-8798 Japan

**Keywords:** Normal tension glaucoma, Primary open-angle glaucoma, Stereo fundus camera, Disc Damage Likelihood Scale, Optic nerve head

## Abstract

**Purpose:**

The Glaucoma Stereo Analysis Study (GSAS), a cross-sectional multicenter collaborative study, used a stereo fundus camera (nonmyd WX) to assess various morphological parameters of the optic nerve head (ONH) in glaucoma patients. We examined the associations between the Disc Damage Likelihood Scale (DDLS), a grading system for estimating glaucomatous ONH damage, and each parameter.

**Methods:**

The study included 187 eyes of 187 patients with primary open-angle glaucoma or normal-tension glaucoma. ONH morphological parameters including the DDLS stage were calculated with prototype analysis software. Three independent graders classified each optic disc appearance into four different types: focal ischemic, myopic glaucomatous, senile sclerotic, and generalized enlargement. The correlations between the DDLS and patient characteristics or each ONH parameter were analyzed with Spearman’s rank correlation coefficient.

**Results:**

The DDLS was correlated positively with baseline intraocular pressure and visual field pattern standard deviation, and negatively with visual field mean deviation. The DDLS was strongly correlated with vertical cup-to-disc ratio and horizontal cup-to-disc ratio positively, and with minimum rim-disc ratio negatively. The mean DDLS stage in the myopic glaucomatous type tended to be higher than the scores in other types.

**Conclusion:**

The DDLS obtained through three-dimensional ONH analysis correlates well with the severity of glaucomatous ONH and visual field damage.

## Introduction

Glaucomatous optic neuropathy (GON) is characterized by nerve fiber loss that can be recognized as thinning of the neuronal rim and enlargement of the excavation in the optic nerve head (ONH). The cup-to-disc (C/D) ratio is a well-known method to estimate the degree of excavation [1], but this ratio does not consider optic disc size. In a previous study using human donor eyes, the optic nerve fiber count increased significantly with enlarging optic disc size [2]. The Disc Damage Likelihood Scale (DDLS) is a method for estimating the degree of optic nerve damage which reflects disc size and has high intraobserver and interobserver reproducibility [3, 4]. The DDLS divides discs into three sizes, small (<1.5 mm), average (1.5–2.0 mm), and large (>2.0 mm), and is based on the width of the neuronal rim or the circumferential extent of absence of the neuronal rim [3, 4].

Although disc size is important for estimating GON damage, another pivotal factor for understanding GON damage is the ONH type. A previous study classified the glaucomatous ONH into four groups: focal ischemic (FI), myopic glaucomatous (MY), senile sclerotic (SS), and generalized enlargement (GE) [5]. Because the speed of progression of glaucomatous visual field defects may differ with each type [6, 7], identifying the type may be useful for predicting disease progression.

Topographic analysis with a simultaneous stereo fundus camera (nonmyd WX, Kowa Company, Ltd., Japan) is a noninvasive, noncontact imaging technique. The Glaucoma Stereo Analysis Study (GSAS) is a multicenter study using this technique to assess various morphological parameters of the ONH in Japanese glaucoma patients. The GSAS has recently demonstrated that significant negative associations were observed between the vertical C/D ratio and visual field mean deviation (MD) and between the disc tilt angle and refractive error [8]. In the present phase of the GSAS, we examined the relationships between the DDLS stage and patient characteristics or various ONH parameters and compared the mean DDLS stage among the four different disc types.

## Patients and methods

The GSAS is a cross-sectional, multicenter, collaborative study, and we have recently reported the basic data including patient characteristics and representative ONH parameters [8]. It was approved by the institutional review boards of the Tohoku University Graduate School of Medicine, Shimane University Faculty of Medicine, Fukui-ken Saiseikai Hospital, Sapporo Teishin Hospital, and St. Marianna University School of Medicine. All experimental procedures were conducted in accordance with the tenets set forth in the Declaration of Helsinki. For this type of study, hospital-based and retrospective, formal consent is not required. All data collected from the participating institutions were analyzed anonymously.

One hundred and eighty-seven eyes of 187 patients with normal-tension glaucoma or primary open-angle glaucoma, comprising 100 men and 87 women aged (mean ± standard deviation) 61 ± 9 years, were recruited into this study from five institutions: Tohoku University Hospital, the Hospital of Shimane University Faculty of Medicine, Fukui-ken Saiseikai Hospital, Sapporo Teishin Hospital, and the Hospital of St. Marianna University School of Medicine, as previously reported [8]. Briefly, the patients underwent full clinical ophthalmologic evaluation, including testing for refractive error and intraocular pressure (IOP) with Goldmann applanation tonometry, as well as slit lamp and fundus examinations. At least one measurement of pretreatment IOP (baseline IOP) was obtained retrospectively. Presurgical data on refractive error was also collected from eyes that had undergone refractive procedures such as cataract surgery. Visual field examinations with the Humphrey visual field analyzer (HFA; Carl Zeiss Meditec Inc., Dublin, CA, USA) were performed on all subjects within 6 months of recruitment. Data from at least six HFA examinations performed were also collected retrospectively for each patient. Additional inclusion criteria included: 1) best corrected visual acuity of 0.155 or better (LogMAR); 2) no congenital ONH anomalies; 3) ONH size within the typical normal range, defined as a disc-macula distance to disc diameter (DM/DD) ratio between approximately 2.4 and 3.0, 4, no clinically apparent secondary cause of glaucoma, and no other disease affecting the visual field; 5) no history of intraocular surgery other than cataract or glaucoma surgery; 6) no history of cataract or glaucoma surgery in the previous three years; and 7) glaucomatous visual field loss more than −12 dB MD. If both eyes met the inclusion criteria, the eye with more advanced glaucoma was selected [8].

The stereo pair of ONH photographs was obtained with a simultaneous stereo fundus camera (nonmyd WX). The nonmyd WX produces nonmydriatic fundus stereographs, and the built-in software (VK-2 WX, prototype version, Kowa Company, Ltd., Japan) automatically calculates ONH morphological parameters and the DDLS stage (nine stages: 0a, 0b, 1, 2, 3, 4, 5, 6, 7) based on manually set contour lines for the ONH disc and cup, which in this study were determined by one of the authors (M.T.) while viewing the images stereoscopically. This determination was made according to the recommendations of the Japan Glaucoma Society Guideline for Glaucoma, 3rd edition, as previously reported [8].

Three independent assessors (T.N., K.O., and Y.Y.) classified each optic disc appearance into four different types: 1) FI discs showing localized tissue loss at the superior or inferior poles and a relatively intact neuroretinal rim elsewhere; 2) MY discs that had a tilted appearance and temporal crescent peripapillary atrophy (PPA) accompanied by additional evidence of glaucomatous damage, excluding discs with degenerative myopia; 3) SS discs with a saucerized shallow cup and diffuse neuroretinal rim tissue loss accompanied by surrounding PPA and choroidal sclerosis; and 4) GE discs characterized by a diffusely enlarged round cup and lack of localized defects of the neuroretinal rim with a previously reported Nicolela’s clarification [5]. Discs that had features of multiple disc types were assigned to the most prominent one. If there was disagreement on the ONH classification among the three assessors, a consensual decision was adopted.

### Statistical analysis

Statistical analysis was performed with JMP pro 10.02 (SAS Institute Inc., Cary, NC, USA) for Windows. Continuous variables were expressed as mean values ± standard deviation. The Spearman rank correlation coefficient was used to determine correlations between patient characteristics and the ONH parameters obtained. In this analysis, ordinal data were treated as continuous. The level of significance was 0.05 in all statistical tests.

## Results

**Fig. 1:**
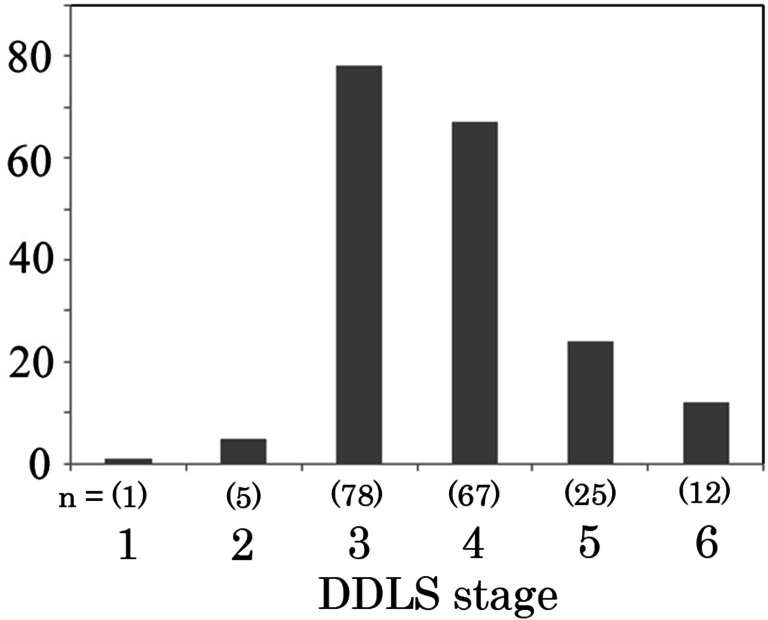
Histogram of the distribution of the DDLS stage in patients in this study. DDLS = Disc Damage Likelihood Scale

A histogram of the distribution of the DDLS stage in the 187 patients is shown in Fig. 1. The average DDLS stage was 3.77 ± 0.95. The relationships between DDLS and patient characteristics are shown in Table 1. There was a weak, but significant positive correlation between the DDLS stage and baseline (pretreatment) IOP (*r* = 0.150, *p* = 0.040, Table 1) and a negative correlation between the DDLS stage and spherical equivalent refractive errors on both the test day (*r* = −0.150, *p* = 0.041) and pretreatment (*r* = −0.183, *p* = 0.012, Table 1). Consistent with the findings in a previous study [3], a significant negative correlation was observed between the DDLS stage and MD (*r* = −0.267, *p* < 0.001, Table 1) and a positive correlation was observed between the DDLS stage and visual field pattern standard deviation (PSD) (*r* = 0.233, *p* = 0.001, Table 1). The relationships between the DDLS and ONH parameters are shown in Table 2. Among the ONH morphological parameters, there were moderate positive correlations between the DDLS and vertical C/D ratio (*r* = 0.381, *p* < 0.001, Table 2) and between the DDLS and horizontal C/D ratio (*r* = 0.292, *p* < 0.001, Table 2). In agreement with those findings, there was a strong negative correlation between the DDLS and minimum rim-to-disc (R/D) ratio (*r* = −0.659, *p* < 0.001, Table 2). Similarly, there was a significant positive correlation between the DDLS and C/D area ratio (*r* = 0.292, *p* < 0.001, Table 2) and a moderate negative correlation between the DDLS and R/D area ratio (*r* = −0.361, *p* < 0.001, Table 2).

**Table 1:** Correlation between DDLS stage and glaucoma patient characteristics

Patient data				
DDLS	Age (years)			
	*r*	Lower 95 % CI	Upper 95 % CI	*p* value
	0.032	−0.112	0.175	0.666
	Corneal curvature radius on the test day (mm)			
	−0.096	−0.236	0.048	0.191
	Spherical refrative error on the test day (D)			
	−0.156	−0.293	−0.013	0.033
	Pretreatment spherical refractive (D)			
	−0.150	−0.324	−0.007	0.041
	Pretreatment spherical equivalent refractive error (D)			
	−0.183	−0.318	−0.040	0.012
	Pretreatment IOP (mmHg)			
	0.150	0.007	0.288	0.040
	MD (dB)			
	−0.267	−0.395	−0.128	<0.001
	PSD (dB)			
	0.233	0.093	0.364	0.001
	Anti-glaucoma eye drops (Number)			
	0.150	0.007	0.287	0.040

*DDLS* Disc Damage Likelihood Scale, *MD* mean deviation, *PSD* pattern standard deviation

**Table 2:** Correlation between DDLS stage and ONH parameters

	Optic nerve head parameters			
DDLS	Vertical cup-disc ratio			
	*r*	Lower 95 % CI	Upper 95 % CI	*p* value
	0.381	0.251	0.497	<0.001
	Horizontal cup-disc ratio			
	0.292	0.155	0.418	<0.001
	Minimum rim-disc ratio			
	−0.659	−0.733	−0.570	<0.001
	Superior minimum rim-disc ratio			
	−0.242	−0.373	−0.102	0.001
	Angle of superior minimum rim-disc ratio			
	−0.167	−0.303	−0.024	0.022
	Inferior minimum rim-disc ratio			
	−0.528	−0.625	−0.417	<0.001
	Superior rim width			
	−0.240	−0.371	−0.100	0.001
	Inferior rim width			
	−0.363	−0.481	−0.231	<0.001
	Rim area			
	−0.471	−0.576	−0.352	<0.001
	Cup-disc area ratio			
	0.366	0.235	0.484	<0.001
	Rim-disc area ratio			
	−0.361	−0.480	−0.230	<0.001

*DDLS* Disc Damage Likelihood Scale, *ONH* optic nerve head

Three independent assessors classified the 187 patients into four groups: FI (*n* = 34), GE (*n* = 38), MY (*n* = 96), and SS (*n* = 19). The distribution of the DDLS stage in the four groups is shown in Table 3, and the mean DDLS stage in Table 4. The degrees of pretreatment spherical equivalent refractive errors in MY and SS were markedly less than those in FI and GE (Table 4). There was a significant difference in the mean DDLS stage between FI and MY (3.53 and 3.91, respectively, *p* = 0.0482, Table 4). However, there was no significant difference in MD or baseline IOP among the four groups (Table 4). In addition, there was no significant difference in PSD between FI and MY, although it in GE was the least compared with those in the other groups (Table 4).

**Table 3:** Distribution of DDLS stage in each disc type group

DDLS	FI	GE	MY	SS	
1	0	0	1	0	1 (0.5 %)
2	0	1	2	2	5 (2.7 %)
3	19	16	37	6	78 (41.7 %)
4	12	16	32	7	67 (35.8 %)
5	3	4	13	4	24 (12.8 %)
6	0	1	11	0	12 (6.4 %)
	34 (18.2 %)	38 (20.3 %)	96 (51.3 %)	19 (10.2 %)	187 (100 %)

*DDLS* Disc Damage Likelihood Scale, *FI* focal ischemic, *GE* generalized enlargement, *MY* myopic glaucomatous, *SS* senile sclerotic

**Table 4:** Demographic data in each disc type group

	FI	GE	MY	SS
No. of patients	34	38	96	19
Baseline IOP (mmHg)	16.1 ± 4.9	17.6 ± 3.9	16.8 ± 3.5	18.0 ± 7.0
Pretreatment spherical equivalent refractive error (D)	−0.75 ± 2.48	−0.98 ± 3.21	−5.18 ± 2.97	−3.77 ± 4.89
	*p* < 0.0001 vs. MY	*p* <0.0001 vs. MY	*p* <0.0001 vs. FI and GE	*p* <0.005 vs. FI and GE
	*p* <0.005 vs. SS	*p* <0.005 vs. SS		
Mean deviation (dB)	−4.51 ± 2.80	−4.38 ± 3.71	−4.96 ± 3.30	−4.44 ± 2.95
Pattern standard deviation (dB)	8.80 ± 4.07	6.39 ± 4.09	8.71 ± 4.18	6.99 ± 3.63
	*p* <0.05 vs. GE	*p* <0.05 vs. FI and MY	*p* <0.05 vs. GE	
Mean DDLS ± SD	3.53 ± 0.66	3.68 ± 0.81	3.91 ± 1.08	3.68 ± 0.95
	*p* <0.05 vs. MY		*p* <0.05 vs. FI	

Data was expressed as mean values ± standard deviation. Statistical significance was tested by ANOVA. *FI* focal ischemic, *GE* generalized enlargement, *MY* myopic glaucomatous, *SS* senile sclerotic, *IOP* intraocular pressure, *DDLS* Disc Damage Likelihood Scale

## Discussion

In optical coherence tomography (OCT) measurements, a fixed-diameter circular scan shows the difference in the distance between the scan point and the ONH margin dependent on the disc size, i.e., a smaller disc may have a long distance leading to a thinner retinal nerve fiber layer (RNFL) thickness, and a larger disc may display a thicker RNFL thickness. Therefore, one must be careful in interpreting the findings between disc size and RNFL thickness from OCT studies. Nonetheless, two different groups reported that RNFL thickness significantly increased with an increase in optic disc size [9, 10]. It was also reported that the Heidelberg Retina Tomograph (HRT) sensitivity required the definition of optic disc size classes or statistical correction for the size of the optic disc [11]. However, a recent study using the HRT II with the corrected effects of magnification on the disc measurements has shown that there was no significant association between RNFL thickness and optic disc area and suggested that the link between RNFL thickness and apparent disc size in the OCT study was probably due to magnification artifacts [12]. Although this does not support the assumption that this staging system reflects disc size, a human histological study that used a relatively high number of eyes (72 eyes from 56 donors) found increasing axon numbers with greater optic disc size [2], suggesting that the DDLS yields accurate estimates.

The DDLS is more reproducible than the C/D ratio system of estimating the amount of disc damage in patients with glaucoma [3, 4] and has been reported to be useful. For example, it was shown that the DDLS significantly correlated with all global and sectoral visual field indexes and with sectoral rim area HRT II measurements [13]. That study also showed that the DDLS stage correlated most strongly with superior and inferior regional data from HRT II and visual field measurements, and less well with temporal and nasal data [13]. The same group also demonstrated that the DDLS had the highest area under the curve (AUC) of 0.91 for predictive values of variables in differentiating glaucomatous from suspect or normal eyes rather than other factors including vertical C/D ratio (AUC = 0.81), MD (AUC = 0.78), HPA scoring (AUC = 0.75), HRT II rim area (AUC = 0.62), and Moorfield’s regression analysis (AUC = 0.54) [14]. In the present study, the DDLS stage was obtained automatically with the prototype analysis software. Using the same system, Han et al. have recently found good agreement (weighted kappa value, 0.59 ± 0.03) between DDLS stages obtained by stereo photography (nonmyd WX) and a glaucoma specialist [15]. There are some comparable findings between their study and our current study. For example, our present data show that the average DDLS stage was 3.77 ± 0.95 (all glaucoma patients, *n* = 187), whereas it was 4.23 ± 1.23 in glaucoma patients in their study (*n* = 80) [15]. Because the average MD was −4.71 ± 3.26 dB and −8.57 ± 8.78 dB in our study and theirs, respectively, their study included more patients with severe glaucoma. It should be noted that our criteria for patient enrollment only included MD > −12 dB, as previously reported [8].

In the present study, there was a significant positive correlation between the DDLS stage and baseline IOP. This is consistent with the previous finding that progression is associated with the level of baseline IOP [16]. In addition, consistent with the results of previous studies [3, 17], we also found that the DDLS stage was significantly inversely correlated with the MD and positively correlated with the PSD, although the correlations were weak to moderate. These findings suggest that the DDLS stage reflects the degree of visual field damage that is measured and expressed by MD and PSD. Since the DDLS is based on the width of the neuronal rim or the circumferential extent of the absence of the neuronal rim, it is reasonable to find that the DDLS was correlated with both the vertical C/D and horizontal C/D ratios. This is in agreement with the findings from OCT data in a previous study [18]. That study also suggested that the DDLS was more useful than any other parameter acquired by OCT [18]. Furthermore, we demonstrated that the DDLS stage has a good inverse correlation with the minimum R/D ratio. Taking these results together, it is likely that the DDLS reflects the degree of ONH damage accurately.

Another noteworthy finding is the comparison of DDLS stages among the four different disc types. Myopia is a known risk factor for glaucoma in Asian [19, 20] and Latino [21] populations. A systematic review and meta-analysis demonstrated that individuals with myopia have an increased risk of developing OAG [22]. We found a negative correlation between the DDLS stage and spherical equivalent refractive errors. Therefore, one possibility is that myopia leads to a high degree of ONH damage. Among the glaucomatous ONH types classified using Nicolela’s system, we found a significant difference in the mean DDLS stage between the FI and MY groups. MY patients had displayed significantly higher mean DDLS stage than FI patients. Another possibility is the overestimation of the DDLS in MY due to the slope in the temporal side. We found no significant differences in the mean MD and PSD between the FI and MY groups, although the MY group tended to have a higher mean MD value (MY, −4.96 ± 3.30 dB; FI, −4.51 ± 2.80 dB). To our knowledge, this is first study demonstrating the relationship between the DDLS stage and different glaucomatous ONH types.

One limitation of topographic analysis with a simultaneous stereo fundus camera is that it is not entirely automated software, and there might still be some bias caused by arbitrarily set margins in discs with atypical shapes, such as myopic discs with temporal crescent PPA or saucerized discs with shallow cupping, where it is difficult to define the margins. Nonetheless, determining the margins is more accurate in stereoscopic images than in monoscopic images.

In conclusion, the DDLS stage obtained through stereoscopic analysis was well correlated with ONH parameters and visual field damage. The DDLS tends to be categorized into higher stages in eyes with myopic disc appearance.
